# Advances in the application of temporal interference stimulation: a scoping review

**DOI:** 10.3389/fnhum.2025.1536906

**Published:** 2025-05-30

**Authors:** Jiaojiao Peng, Zhanhong Du, Yi Piao, Xianxian Yu, Kaiqi Huang, Yan Tang, Pengfei Wei, Pu Wang

**Affiliations:** ^1^Department of Rahabilitation Medicine, The Seventh Affiliated Hospital of Sun Yat-Sen University, Shenzhen, China; ^2^The Brain Cognition and Brain Disease Institute (BCBDI), Guangdong Provincial Key Laboratory of Brain Connectome and Behavior, CAS Key Laboratory of Brain Connectome and Manipulation, Shenzhen-Hong Kong Institute of Brain Science, Shenzhen Institute of Advanced Technology, Chinese Academy of Sciences, Shenzhen, China; ^3^Faculty of Life and Health Sciences, Shenzhen University of Advanced Technology, Shenzhen, China; ^4^Shenzhen Fundamental Research Institutions, Shenzhen, China; ^5^University of Chinese Academy of Sciences, Beijing, China; ^6^Shenzhen Zhongke Huayi Technology Co., Ltd., Shenzhen, China; ^7^School of Biological Science and Medical Engineering, State Key Laboratory of Digital Medicine, Southeast University, Nanjing, China

**Keywords:** temporal interference stimulation (TIS), neuromodulation, brain stimulation, deep brain stimulation (DBS), transcranial electrical stimulation (TES), disorders

## Abstract

**Introduction:**

Neuromodulation is an emerging technology that combines biomedical engineering and neuroscience to modulate the nervous system using implantable or non-implantable devices, which have proven effective in treating neurodegenerative and neuropsychiatric disorders. There is an urgent need to develop a noninvasive deep brain stimulation technique that combines the advantages of safety, non-invasiveness, and precise deep brain stimulation to address the invasiveness and lack of focus of existing neuromodulation techniques.

**Objective:**

Our primary goal is to better understand the progress of research on the application of temporal interference stimulation (TIS).

**Methods:**

A scoping review was conducted following Arksey and O'Malley’s methodological framework, utilizing the databases PubMed, Web of Science, and EMbase. Original research of any study design, focused on the topic and published in English from the inception of each database until June 2024, was included.

**Results:**

A total of 708 studies were identified in the databases, with 33 studies ultimately included. The literature primarily addresses the development and application of TIS. All studies demonstrate that TIS can effectively target deep areas of the brain.

**Conclusion:**

TIS can effectively penetrate the cerebral cortex and modulate neural activity in deep brain regions. Additionally, TIS shows potential for treating a wide range of central nervous system (CNS) disorders, though the underlying mechanisms remain unknown. This scoping review provides a series of recommendations to guide future research in exploring the applications of TIS.

## 1 Introduction

Neuromodulation has emerged as a promising diagnostic and therapeutic technology over the past few decades (Denison and Morrell, 2022). This technology integrates biomedical engineering and neuroscience to modulate the nervous system using implantable or non-implantable devices (Du et al., 2020; Li et al., 2023). Neuromodulation employs physical methods (such as light, electricity, magnetism, or ultrasound) or chemical approaches (such as drugs) to stimulate neurons, producing inhibitory or excitatory effects that can alleviate clinical symptoms, enhance neurological function, and ultimately improve quality of life (Du et al., 2015; Du et al., 2017a). Due to its effectiveness in treating neurodegenerative and neuropsychiatric disorders, neuromodulation has received extensive global attention.

Invasive and noninvasive brain stimulation techniques are important modalities for neuromodulation techniques (Shukla and Thirugnanasambandam, 2021; Sha et al., 2023; Sha and Du, 2024). Commonly used physical stimulation techniques in clinical practice include transcranial magnetic stimulation (TMS), transcranial electrical stimulation (tES), and deep brain stimulation (DBS) (Adair et al., 2020). DBS is one of the most advanced forms of invasive neuromodulation, involving the implantation of stimulating electrodes into deep brain structures to precisely target brain nuclei and regulate brain circuitry dynamics (Du et al., 2017b; Krishnan et al., 2021; Parker et al., 2021). As a highly focused and controlled neuromodulation approach, DBS has been widely applied to treat neurological disorders that are resistant to conventional therapies, including Parkinson’s disease, tremors, and dystonia (Lozano et al., 2019). However, DBS carries risks such as brain hemorrhage and infection, which may limit its practical application.

Unlike DBS, TMS and tES are two noninvasive techniques commonly used in clinical practice and have been widely used in clinical research in recent decades (Begemann et al., 2020). TMS and tES can be used to treat epilepsy, stroke, schizophrenia, and depression by applying electrical or magnetic forces to the human scalp via coils or electrodes, resulting in acute and neuroplastic changes in cortical excitability (Camacho-Conde et al., 2022; Rawji et al., 2020). These noninvasive techniques have the advantages of being safe, tolerable, cost-effective, and easy to administer. However, the effects of noninvasive brain stimulation via TMS and tES on neurons are variable and difficult to assess. Furthermore, due to the complex structure of the human brain, magnetic and electrical signals exhibit absorption and scattering properties within brain tissue, and electric and magnetic fields typically decrease dramatically with depth, resulting in low spatial resolution for most noninvasive brain stimulation modalities (Yavari et al., 2018).

Therefore, there is an urgent need to develop a noninvasive deep brain stimulation technique that combines the advantages of safety, noninvasiveness, and precise deep brain stimulation to address the invasiveness and lack of focus of existing neuromodulation techniques (Caulfield and George, 2018; Liu et al., 2021). Grossman et al. (2017) proposed a new noninvasive brain-stimulating technique called “temporal interference” (TI) electrical stimulation. TI stimulation is based on the simultaneous application of two high-frequency sinusoidal currents (≥1 kHz) with slightly different frequencies to produce TI patterns. It is well-known that neurons do not respond to high frequency sinusoidal current stimulation (Hutcheon and Yarom, 2000). However, if two high-frequency sinusoidal electric fields with a slight frequency difference (such as 2 kHz and 2.01 kHz) are applied simultaneously, the low-frequency envelope modulation signal (such as 10 Hz) formed by the superposition of the two electric fields in the deep brain, which is close to the frequency of the brain’s endogenous neural rhythms, can effectively modulate the neural activities in the deep brain regions (Dmochowski and Bikson, 2017). Because of its ability to stimulate deep pathogenic areas, TIS has potential for the treatment of neurological disorders. However, this technology is still in its early stages and there are several ongoing efforts for further investigation in computational models, animal studies, and human trials (Bouthour et al., 2017). To inform future research and further the development and application of TIS, this paper reviews the current research progress in the application of TIS.

## 2 Methods

### 2.1 Scoping review

For this scoping review, we use the method logical framework developed by Arksey and O’Malley (Arksey and O'Malley, 2005). Arksey and O’Malley suggest that there are five stages to a scoping review: (1) identifying the research question; (2) identifying relevant studies; (3) selecting studies; (4) charting the data; (5) collating, summarizing, and reporting the results. This scoping review is conducted following the Preferred Reporting Items for Systematic Reviews and Meta-Analyses (PRISMA) guidelines for scoping reviews (Tricco et al., 2018).

### 2.2 Identifying the research question

In this review, we will concentrate on research advances in TIS, understanding of its physiological mechanisms, development and application, as well as stimulation optimization of TIS. This scoping review aims to guide the use of TIS as a noninvasive neuromodulation therapeutic tool.

### 2.3 Identifying relevant studies

PubMed, Web of Science, and Embase databases were searched from their inception until June 2024. In addition, reference lists of relevant articles were screened to identify key articles that may have been missed. The following search terms were entered using the Boolean operators AND/OR: “temporal interference”; “temporally interfering.”

### 2.4 Selecting studies

Inclusion and exclusion criteria were formulated. The inclusion criteria encompassed: (1) studies involving animal models or human subjects; (2) the use of TIS as an intervention; (3) research focused on the potential application of TIS in disease contexts; and (4) publications written in English. The exclusion criteria were: (1) conference abstracts, systematic reviews, and meta-analyses; and (2) studies for which, despite efforts to retrieve the paper, the article was either withdrawn or the full text was inaccessible.

### 2.5 Data management, screening, and extraction

**Figure 1:**
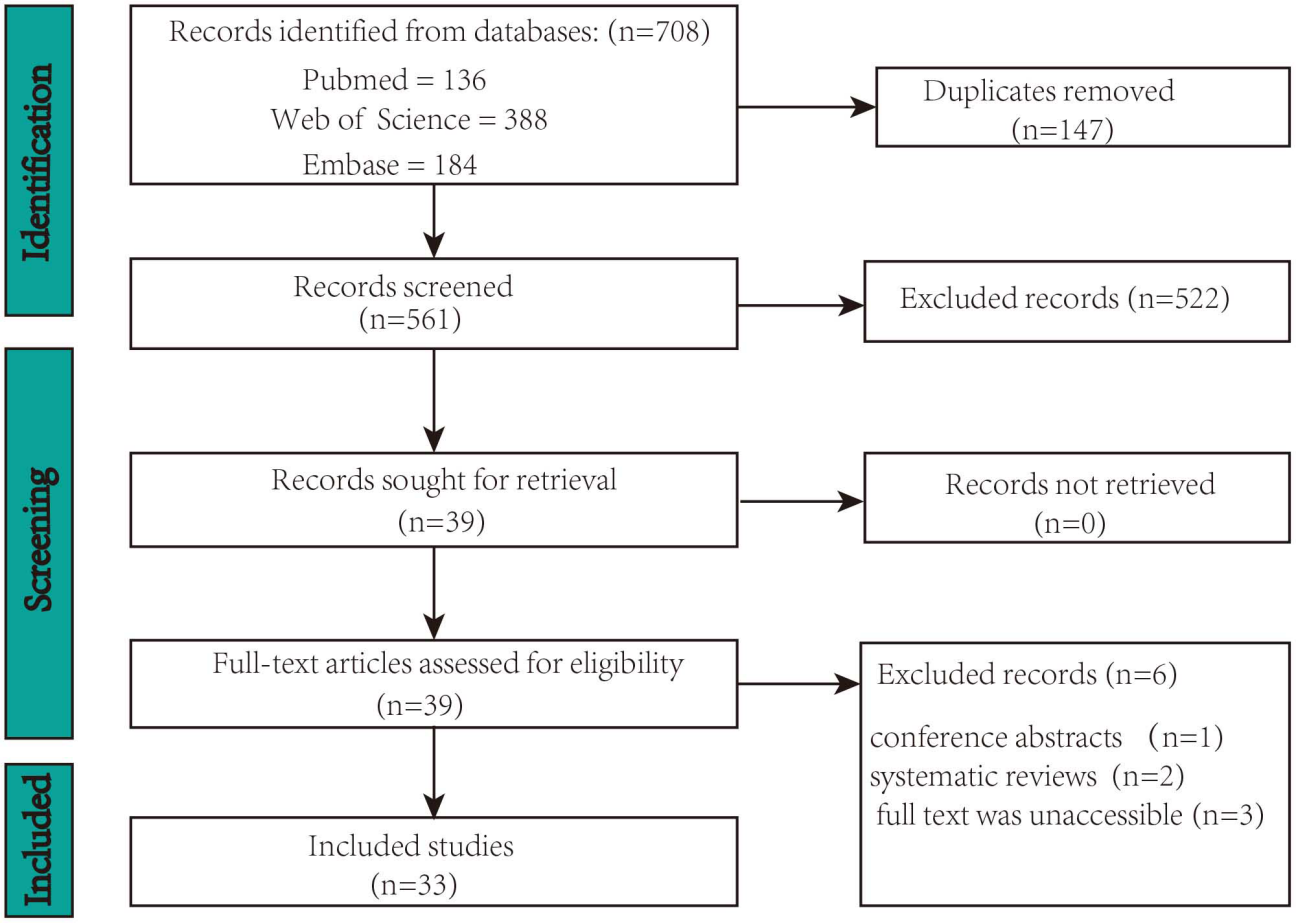
PRISMA flowchart for the selection of studies.

The following phases were involved in study selection: titles and abstracts were reviewed for relevance by two reviewers according to the inclusion and exclusion criteria above. Full-texts were then screened. The two authors reached a consensus to determine whether this study should be included or excluded. If the two authors failed to reach a consensus, a third author would have been consulted. Included articles were then examined to extract data. The process of identification, screening, eligibility, and inclusion of studies is pictured in Figure 1.

### 2.6 Data collection and synthesis

A data extraction form will be employed to collect information from the identified literature, in line with the objectives of the proposed scoping review. The extracted data will comprise bibliographic details including the first author, year, title, study population, target area and outcomes. To evaluate whether TI stimulation deeply modulated the target area, we extracted the changes observed before and after stimulation. The extracted data will be presented in tabular format to address the review questions, as recommended by the JBI guidelines for scoping review protocols (Peters et al., 2021).

### 2.7 Collating, summarizing, and reporting the results

**Figure 2:**
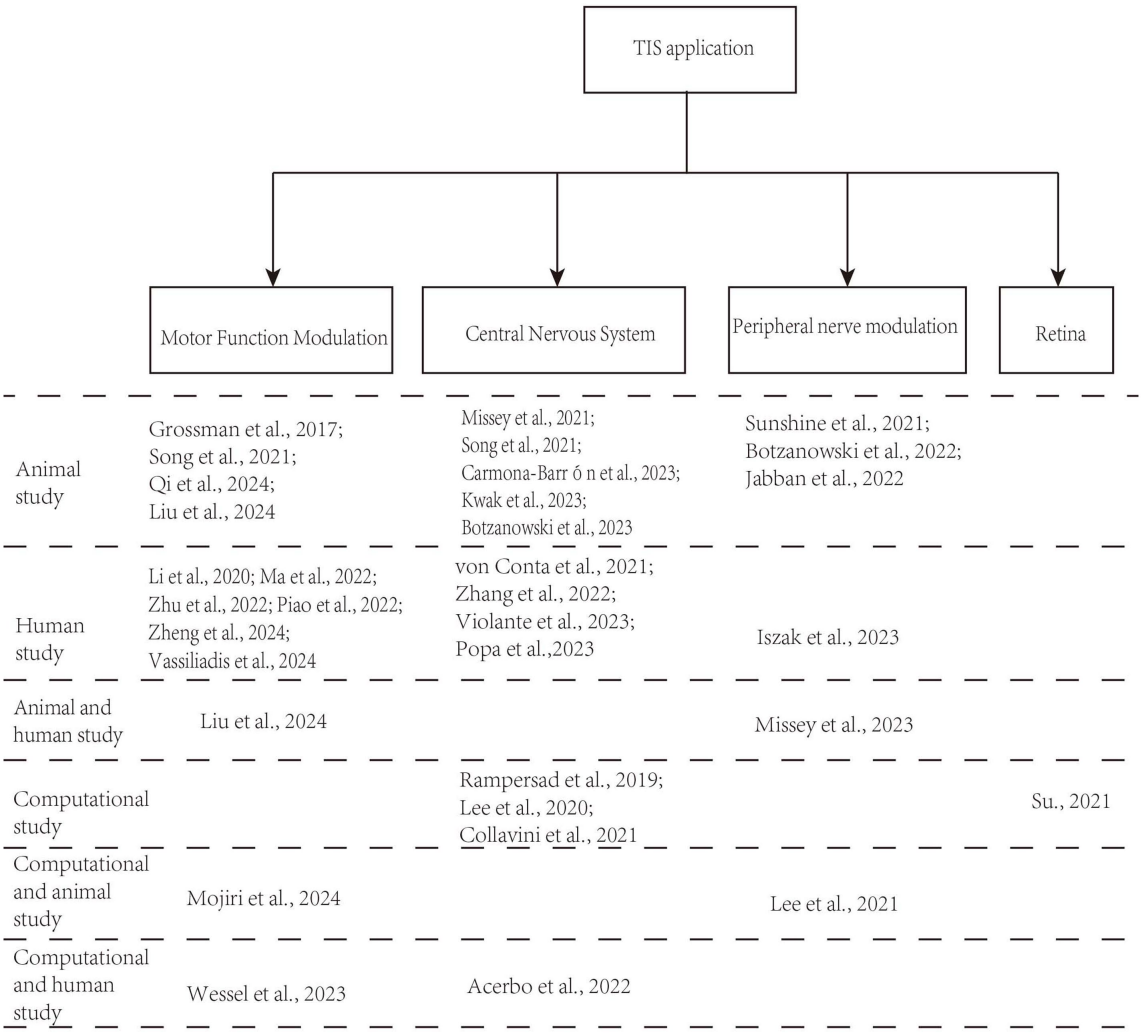
The organization of included studies based on TIS application.

A total of 708 records were identified in the three databases. Of these, 147 duplicate records were eliminated. Ultimately, 33 records met the inclusion criteria of the screening procedure. Figure 1 displays the screening procedure and the rationales for excluding research. The research findings are summarized in Supplementary Table S1 and organized in Figure 2 based on TIS application.

## 3 Search results

The flowchart in Figure 1 summarizes the study selection process. A total of 708 studies were initially identified. Subsequently, 147 duplicate studies were removed, leaving 561 studies for screening. Subsequently, 522 studies were excluded based on their titles and abstracts. Among the 39 remaining studies, 6 that did not meet the selection criteria were removed after a full-text review. Consequently, 33 articles were included in the review.

## 4 Discussion

### 4.1 Origins and principles

**Figure 3:**
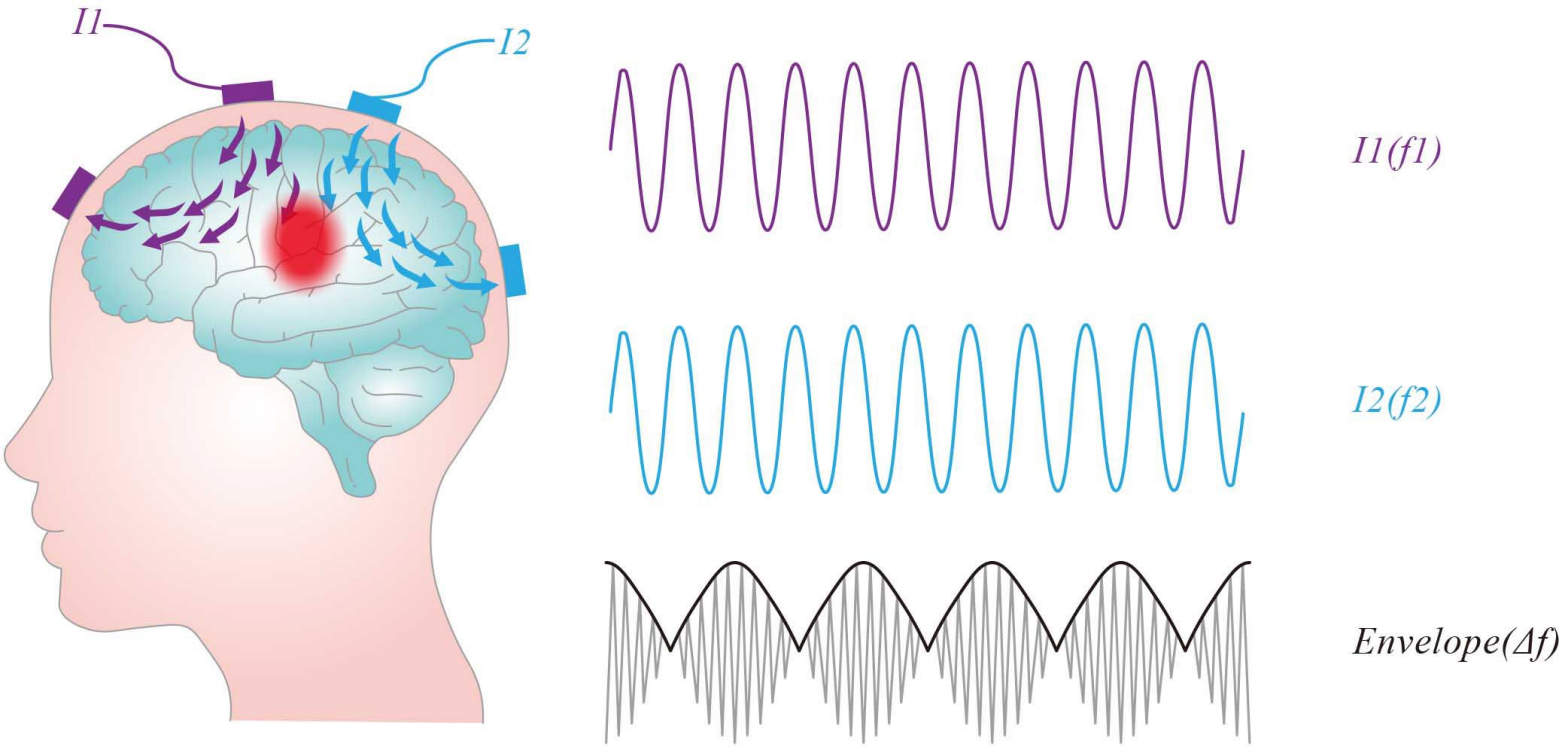
Concept of temporal interference stimulation. Two pairs of stimulation electrodes are attached to the scalp, each supplying an oscillating current and producing an oscillating electric field, I1 (purple line) and I2 (blue line). The intersection of the two fields produces an amplitude-modulated field with an envelope that oscillates at the beat frequency Δf (f1-f2).

Neurons exhibit low-frequency characteristics, where only direct current (DC) or low-frequency alternating current (AC) can induce hyperpolarization or depolarization, leading to the generation of action potentials. In contrast, Neurons do not directly respond to high frequency oscillating (e.g., ≥1 kHz) electric fields (Hutcheon and Yarom, 2000). In the 1950s, Austrian scientist Hans Nemen proposed the use of interferential currents (IFC) as a method of electrical stimulation therapy for peripheral stimulation (Goats, 1990). When two slightly different frequencies (f₁ and f₂) of sinusoidal alternating current interact, they can result an amplitude-modulated field with an envelope that oscillates at the beat frequency Δf (f1-f2). This stimulation method is named TI stimulation, which is shown in Figure 3. For instance, when high-frequency alternating currents of 2000 Hz and 2010 Hz intersect, a low-frequency envelope waveform at 10 Hz would be produced. TI stimulation delivers high-frequency currents that can penetrate deep human tissue while concurrently producing an envelop modulation at a site deep in the brain, which can modulate deep-lying neurons without recruiting overlying ones. Additionally, TI stimulation allows for the delivery of greater current to deep tissues without surpassing the pain thresholds of the skin.

Traditional tES faces limitations in its ability to penetrate brain cortex using low-frequency electricity. The area of neural stimulation remains proximal to the electrodes in the cerebral cortex, making it challenging to achieve the depth of stimulation that traditional implantable electrodes can reach. However, by applying two high-frequency electrical stimulation with slightly different frequencies to the brain, a low-frequency envelope electric field forms at the intersection of the two currents, enabling the modulation of deep-brain neurons.

The principle of temporal interference was innovatively applied to the brain by Grossman et al., who introduced the concept of TIS (Grossman et al., 2017). In their experiments with mice, they demonstrated that neurons responded to the frequency difference between two high-frequency electrical stimuli of similar frequencies after interference, with TIS inducing neuronal firing in deep brain regions, such as the hippocampus, while cortical neurons remained inactive. This study provides compelling evidence that TIS can effectively activate neurons in the hippocampal region, allowing for more precise modulation of deep brain regions through the adjustment of stimulation parameters. This study represents the first successful validation of noninvasive focal stimulation targeting deep brain regions, a significant advancement in the field of brain stimulation. Future research should investigate whether TIS produces effects comparable to other noninvasive neuromodulation techniques, addressing both acute neural activity changes during stimulation and long-term alterations in synaptic plasticity (Yavari et al., 2018).

### 4.2 Mechanisms

The underlying mechanisms of TIS can be categorized into suprathreshold and subthreshold activation paradigms. The dominant mechanism of TIS depends critically on the electric field strength experienced by the neural tissue, with higher field strengths favoring suprathreshold activation and lower field strengths favoring subthreshold modulation. Lee et al. (2020) demonstrated using finite element human head models that maximal hippocampal field strengths reach only 0.38 V/m—orders of magnitude below the ~100 V/m threshold for direct neuronal activatio. In contrast, mouse head models showed the field strengths can achieve 383 V/m at 0.776 mA, confirming suprathreshold stimulation feasibility in rodents but highlighting the impracticality of achieving comparable intensities in humans, where >10 mA would be required—exceeding safety limits. In the application of human experiments of TIS, there are certain difficulties in achieving suprathreshold stimulation. These findings collectively indicate that subthreshold modulation is the primary mechanism in human TIS applications. In order to better elucidate the neuromodulatory effects of TI electrical stimulation, recent studies have independently examined both suprathreshold and subthreshold modulation with TI electrical stimulation.

#### 4.2.1 Suprathreshold activation

The mechanisms underlying suprathreshold activation in neural tissues remain unclear, with two dominant hypotheses emerging from computational and experimental studies.

##### 4.2.1.1 Envelope demodulation hypothesis

Mirzakhalili et al. (2020) first proposed that TI stimulation activate neurons through nonlinear rectification of voltage-gated sodium channels. Their HH simulations suggested that this process extracts low-frequency interference envelopes while blocking high-frequency propagation via sustained sodium channel inactivation. Their model was initially tuned to optimize activation by the TI envelope, aligning with the resonance properties of neurons with nonlinear conduction channels, as discussed by Hutcheon and Yarom (2000). Furthermore, they demonstrated that depending on the spatial distance from the electrodes, neurons could exhibit conduction block, tonic firing, or phasic responses. They showed that nonlinear dynamics of ion channels result in a subthreshold voltage signal containing both the low-frequency envelope (beat frequency) and high-frequency sine wave content—indicating that the high-frequency signals were never blocked. Thus they concluded that the nonlinear dynamics of the axon sort of rectified the signal and extracted the envelope frequency, providing a mechanistic basis for how TI stimulation may engage deep brain targets.

##### 4.2.1.2 Linear integration hypothesis

From another perspective, Budde et al. (2023) reported distinct observations in a study involving live rat sciatic nerves. Using a 12-contact spiral cuff electrode (1.5 mm diameter) with 1.5/1.7 kHz TI protocols (0.3–1 mA), they revealed three key findings: (1) Motor thresholds correlated strongly with peak-to-peak amplitude (R^2^ = 0.93) rather than envelope magnitude; (2) Phase reversal (180°shift) inverted muscle recruitment patterns; and (3) No conduction block occurred up to 10 kHz. These results supported with modified neuron models emphasizing linear summation of subthreshold oscillations through myelinated fiber capacitance, suggesting activation depends on cumulative charge integration rather than envelope extraction. The experimental work by Budde et al. employed a beat frequency of 20 Hz, which was higher than the optimized 5 Hz identified by Mirzakhalili. Thus, it is hypothesized that their axons might not have been recipients of the resonance effects associated with the nonlinear behavior of ion channels. Consequently, their findings were more consistent with linear charging behavior. Notably, their experiments also demonstrated cases of conduction block, as well as tonic and phasic activation—paralleling findings from Mirzakhalili. Phasic firing at the beat frequency was achieved in both HH-based compartmental models and real axons under suprathreshold stimulation, while both experimental and modeling results indicated suboptimal tonic firing and the occurrence of conduction block. Overall, the findings of Budde and Mirzakhalili appear to be complementary. Budde et al. acknowledged that their results did not disprove the “envelope extraction” hypothesis, whereas Mirzakhalili’s work aligns with the threshold charging theory, a mechanism inherently integrated into HH conductance-based circuit models.

##### 4.2.1.3 Translational challenges

Scaling these effects to humans faces critical barriers. Cao and Grover’s (2020) comparative analysis revealed that rodent models achieve 0.38 V/m fields at 0.6 mA in 1 mm nerves (conductivity 0.0346 S/m), while equivalent fields in human gray matter (0.333 S/m) require 9.8 mA—exceeding safety limits by 245%. This explains clinical observations where human hippocampal fields peak at 0.38 V/m, insufficient for direct activation but compatible with subthreshold effects (Lee et al., 2020).

#### 4.2.2 Subthreshold network modulation

Emerging evidence from computational models and experimental studies converge on subthreshold network modulation as a conceivable mechanism of TIS in the central nervous system, though key mechanistic uncertainties remain.

##### 4.2.2.1 Computational insights

Howell and McIntyre (2021) found demonstrated through hybrid axon-neuron models that sodium channel inactivation prevents direct firing at clinically safe currents (≤10 mA). Instead, 2 mA TIS facilitated phase synchronization in cortical networks by entraining subthreshold oscillations—an effect similar to transcranial alternating current stimulation (tACS) but with enhanced spatial specificity. Karimi et al. (2019) further investigated this phenomenon, using detailed axon models. They revealed that human-scale field strengths (<0.4 V/m) fail to demodulate high-frequency carriers (1–10 kHz) through neuronal membranes’ low-pass filtering. Their models showed that axon terminals integrate carrier cycles through capacitive coupling (*τ* ≈ 0.3 ms), generating sustained depolarization only when phase alignment persists for >5 cycles at 200 Hz beat frequencies.

Esmaeilpour et al. (2021) demonstrated that TI stimulation induces subthreshold neuromodulation in humans primarily through amplitude-modulated (AM) fields, which exhibit enhanced intensity in deep brain regions compared to unmodulated cortical field. Through multi-scale computational modeling and experimental validation, they demonstrated that spatial selectivity of TI stimulation arises from phase-dependent modulation of neural oscillations in deep brain structures, facilitated by adaptive network mechanisms (e.g., GABAb receptors) that amplify sensitivity to AM waveforms. Carrier frequency selection critically determines stimulation efficiency: lower carrier frequencies (100 Hz) require weaker scalp currents (~13 mA at 5 V/m) to modulate gamma oscillations, whereas kHz carriers demand impractical currents (>160 mA) due to mismatched neuronal membrane time constants. Sensitivity inversely correlates with membrane response speed—faster time constants reduce required currents. TI selectivity emerges when network adaptation dynamics (e.g., GABAb-mediated inhibition) operate faster than the AM frequency, enabling frequency-specific modulation without suprathreshold activation. Thus, at clinically safe currents (<10 mA), TI predominantly engages subthreshold mechanisms, tuning oscillatory activity through endogenous network properties rather than direct neuronal firing.

##### 4.2.2.2 Experimental validation

Clinical observations provide critical validation for these models. In drug-resistant epilepsy patients, both 1 kHz and 9 kHz TI protocols suppressed hippocampal pathological activity (IEDs/HFOs) without inducing conduction block—contradicting peripheral nerve predictions but aligning with subthreshold entrainment models. The sustained “carry-over effect” (>48 h biomarker suppression) further implicates long-term plasticity mechanisms rather than acute depolarization (Missey et al., 2024). Wireless TI systems using photocapacitors achieved chronic hippocampal theta entrainment (4–8 Hz) in freely moving mice, demonstrating stable modulation over 28 days without tissue damage (Missey et al., 2022). Additionally, cognitive studies revealed dorsomedial prefrontal TI enhances reward-mood coupling through gamma oscillation modulation (Suzuki et al., 2024), while Stroop task improvements correlated with theta-gamma cross-frequency coupling (Ryan et al., 2024). In another study, von Conta et al. (2022) found no significant alpha entrainment (8–12 Hz) despite using theoretically optimal parameters, underscoring methodological challenges in detecting weak subthreshold effects. Additionally, Luff et al. (2024) proposed a pulse-width modulated protocol, which demonstrated that neural membranes convert high-frequency fields (5 kHz) into physiological depolarization through nonlinear capacitance effects—a process exceeding classic low-pass filter predictions by 18%.

### 4.3 Development and applications

As displayed in Figure 2, most contemporary studies investigating the use of TIS have utilized both animal models and human experiments. Researchers have discovered that TIS is effective for motor function, capable of activating muscle nerves for electromyographic stimulation, and holds promise for applications in various central nervous system (CNS) disorders (Esmaeilpour et al., 2021; Sunshine et al., 2021; Vassiliadis et al., 2024a). Additionally, noninvasive TIS has potential applications in other tissues, including the detection of abnormal tissues, the retina, and ocular applications.

#### 4.3.1 Applications of TIS in the motor function modulation

TIS has demonstrated the ability to modulate the motor cortex in both rodent and human brains. Grossman et al. observed periodic movements of the forepaw and whiskers in mice when TI currents were applied to the motor cortex (Grossman et al., 2017). Subsequent studies have further explored the regulatory effects of TI electrical stimulation. For instance, Mojiri et al. (2024) performed a quantitative analysis of noninvasive deep TIS in the HH neuron model of the rat somatosensory cortex and demonstrated that specific parameters of TIS, such as carrier frequency and current range, optimized neuron spiking in model. Experimentally, TIS of the left motor cortex on a rat induced significant contralateral hand movements, confirming the simulation results. Qi et al. (2024) demonstrated that TI electric field brain stimulation significantly enhances motor skills in mice by promoting neuroplasticity. Their study showed that daily TIS of the primary motor cortex (M1) with an envelope frequency of 20 Hz for 7 days improved motor performance. The underlying mechanisms included increased expression of synapse-related proteins, enhanced neurotransmitter release, higher dendritic spine density, and greater synaptic vesicle numbers. Animal and model-based experiments have effectively validated the efficacy of TIS in enhancing motor function. However, its underlying mechanism has rarely been studied. This study is the first to describe its mechanism and reports that TIS can noninvasively improve motor skills by enhancing neuronal excitability and plasticity. As a relatively novel technology, further research should be conducted to uncover the intricate neural principles governing TIS-mediated promotion of motor function. This effort will expedite its facilitate its early translation into clinical practice.

To further explore the effects of TI electrical stimulation on human motor functions, Ma et al. investigated the efficacy of TIS using envelope-modulated waveforms on the human primary motor cortex (M1) in healthy volunteers. The study found that 70 Hz TIS enhanced reaction time and motor cortex excitability, while 20 Hz TIS significantly facilitated motor learning and was positively correlated with increased motor evoked potentials (Ma et al., 2021). Their results represent the validation of the efficacy of TI electrical stimulation on human motor functions and motor cortex excitability. Additionally, stimulation with different envelope frequencies produced varying effects on motor tasks, suggesting frequency-specific modulation by TI electrical stimulation. Subsequent studies have further investigated the regulatory effects of TI electrical stimulation on human motor functions. Zhu et al. (2022) demonstrated that TI electrical stimulation effectively increased the functional connectivity strength between the primary and secondary motor cortex. This increased connectivity contributes to enhance cortical excitability, thereby facilitating the improvement of motor functions. These findings positions TI electrical stimulation as a promising intervention for improving motor learning and facilitating rehabilitation in neurodegenerative disorders such as stroke and Parkinson’s disease. Li et al. (2023) numerically validated the TIS method through simulation experiments and analyzed the effects of different polarities, frequency pairs, and current amplitudes on forearm parameters in five healthy volunteers during selective stimulation (Du et al., 2020). They demonstrated the feasibility of the selective neuromuscular stimulation method by independently activating the nerves/muscles controlling the human fingers using a dual-channel stimulator. Wessel et al. demonstrated that noninvasive theta-burst stimulation of the human striatum via TIS enhances striatal activity and motor skill learning. Using computational modeling, functional magnetic resonance imaging (fMRI), and behavioral evaluations, they found that striatal TIS increased activity in the striatum and motor network, especially improving motor performance in older adults (Wessel et al., 2023). Unlike the methods mentioned before that stimulate the motor cortex, this study focuses on stimulating the striatum. The striatum is not only crucial for motor function but also serves as a key pathophysiological substrate in Parkinson’s disease. This present work is the first to demonstrate, in human subjects, TIS has the capacity to noninvasively modulate neuronal activity within deep brain regions through theta-burst patterned tTIS of the striatum.

Based on the above research, it can be concluded that TI electrical stimulation can effectively enhance motor functions. Nevertheless, the underlying mechanisms remain poorly explored. However, while recognizing the necessity for empirical validation, it should be noted that significant differences still exist between the rodent brain and the human brain, particularly in terms of complex anatomy. Therefore, to validate the effectiveness of TI, it is essential to acquire real data from the human brain under TI stimulation. Further investigations are required to elucidate underlying mechanisms, develop strategies for improving behavioral effects and establish pathways for personalized applications with the ultimate goal of translating this exciting, innovative approach to clinical settings.

#### 4.3.2 Applications of TIS in the central nervous system

By targeting the primary motor cortex (M1) of mice, TI stimulation improved the motor skills of mice by enhancing neuronal excitability and plasticity (Qi et al., 2024). TI electrical stimulation also has the potential to map and stimulate pathological targets (Collavini et al., 2021). In the pre-surgical treatment of drug-resistant epilepsy, Electrical stimulation mapping (ESM) of the brain using stereo-electroencephalography (SEEG) intracranial electrodes, also known as depth-ESM (DESM), is part of the pre-surgical evaluation process to delineate the ‘epileptogenic zone’ (Britton, 2018). Typically, DESM consists in applying the electrical stimulation using adjacent contacts of the SEEG electrodes and in recording the EEG responses to those stimuli. However, the physical location and number of implanted electrodes are constrained by the brain’s complex structure. Consequently, the spatial extension or coverage of the stimulated area is not well defined (Frauscher, 2020). Collavini et al. (2021) proposed using contacts of all SEEG electrodes as an electrode array, rather than the classical adjacent stimulation with only one current source. In the context of tES performed on two realistic head models of real patients undergoing pre-surgical evaluation, by injecting electric currents of different intensities between contacts of electrodes at different depths (denoted as x-DESM), the researchers demonstrated that x-DESM could improve coverage and/or focality without the need to insert additional electrodes. Finally, they show one example of TI stimulation to validate this method. They applied TI electrical stimulation at 10 kHz and 10.01 kHz to two different contact pairs, injecting a current of 1 mA to a realistic head models of real patients undergoing pre-surgical evaluation and observed typical spontaneous seizures. This demonstrates that TI electrical stimulation can achieve focal stimulation in brain regions without the need for additional implanted electrodes, making it useful for precise localization of epileptogenic foci.

It has been shown that the enveloping electric field of TI can be focused on deeper brain regions, potentially aiding epilepsy treatment. To localize the epileptogenic zone, Missey et al. (2021) proposed an orientation-adjustable TI electrical stimulation method. They induced seizure-like events (SLE) in mice using TI electrical stimulation of subdural electrodes. All mice exhibited seizures when 600 μA of TI electrical stimulation was applied to each pair of electrodes. This study demonstrated that the electrophysiological and behavioral events produced by TI electrical stimulation were identical to those produced by implanted electrodes, highlighting the feasibility of this minimally invasive approach. This provides an experimental rationale for using noninvasive TIS to treat epilepsy.

Violante et al. (2023) demonstrated the effectiveness of noninvasive TI electrical stimulation of the human hippocampus. By using electric field modeling and measurements in a human cadaver, they verified that TIS could be focally and steerably targeted to the hippocampus. In order to test whether the stimulating fields could modulate hippocampal neural activity, they applied TI stimulation to 20 healthy participants. Their results showed that TIS modulated hippocampal activity and improved episodic memory accuracy by fMRI and behavioral experiments. The hippocampus is implicated in processes related to learning and memory, spatial navigation, and emotional behavior. It is also associated with numerous brain disorders, including Alzheimer’s disease, schizophrenia, and temporal lobe epilepsy. By noninvasively modulating the neural activity of the hippocampus, TI stimulation presents new opportunities for probing the causal role of the hippocampus in brain functions.

Although human trials of TIS in neurological patients are still in their early stages, early data suggest its potential for clinical translation in nervous system disorders. A pilot trial investigating TIS targeted at the right globus pallidus in patients with mild Parkinson’s disease (PD) has demonstrated significant alleviation of motor symptoms, particularly bradykinesia and tremor, after a single session of transcranial TIS (tTIS) (Yang et al., 2024). Moreover, no severe adverse effects were observed. Similarly, another clinical study applied TIS with an amplitude modulation (AM) frequency of 130 Hz to the hippocampus in patients with mesiotemporal lobe epilepsy (MTLE) (Missey et al., 2024). These patients were implanted with stereoelectroencephalography (sEEG) depth electrodes to investigate changes in epileptic biomarkers following TIS. The results showed that TIS significantly reduced interictal epileptiform discharges (IEDs) and pathological high-frequency oscillations (HFOs). Together, these clinical trials suggest that TIS is a promising noninvasive approach for the treatment of PD and epilepsy.

DBS has long been used to treat Parkinson’s disease, essential tremor, dystonia, and obsessive-compulsive disorder (Lozano et al., 2019), and it shows great potential for treating depression (Malone et al., 2009) and other neuropsychiatric disorders (Casagrande et al., 2019). However, careful wound care and personal hygiene are crucial for protecting DBS hardware and preventing complications after surgery. In contrast, TIS is a novel noninvasive neuromodulation technique capable of delivering focal, steerable stimulation to deep brain areas. Whether TIS can exerts a therapeutic impact on neurological disorders comparable to DBS remains an open question and warrants further investigation. While most TIS studies remain preclinical or computational, preliminary human trials have begun to emerge, primarily focusing on safety and feasibility in both healthy volunteers and patients with milder symptoms. Future research should expand the diversity of patients studied under TI stimulation. Additionally, further investigations into the mechanisms underlying the effects of TI stimulation are needed to enhance our understanding of TI. Overall, TIS presents significant opportunities for future research and is expected to more efficiently regulate brain function.

#### 4.3.3 Applications of TIS in the peripheral nerve modulation

For enhanced therapeutic outcomes, some researchers have proposed combining TIS with peripheral nerve stimulation methods. Sunshine et al. (2021) suggested that TIS may represent a novel approach to stimulating the respiratory system. In their study, they developed a rat model of drug overdose-induced respiratory depression by using TIS with epidural electrodes placed on the rat’s spine. They observed significant diaphragm contractions in the rat model, and breathing quickly resumed when the TIS waveform was adjusted. Furthermore, they discovered that TIS effectively activated spinal motor neurons following spinal cord injury, presenting a new intervention approach for treating spinal cord injuries.

Missey et al. (2023) demonstrated that obstructive sleep apnea (OSA) improves with noninvasive hypoglossal nerve stimulation using TI. They developed a novel form of bilateral TIS for the hypoglossal nerve, showing that it effectively stimulated the nerve at lower amplitudes compared to unilateral TI or traditional transcutaneous stimulation. In human trials, TIS was well-tolerated and reduced apnea-hypopnea events in female patients with OSA. These findings highlight the potential of TI as a safe, effective, and patient-friendly treatment for conditions that require deep nerve stimulation.

Botzanowski et al.’s (2022) study tested TI electrical stimulation on a mouse model of the sciatic nerve, observing significant muscle contractions and leg movements induced by the envelope waveforms, thereby verifying sciatic nerve activation. Lee et al. (2021) employed TI electrical stimulation to treat overactive bladder syndrome, demonstrating that TIS increased urinary output, decreased bladder contraction frequency, and successfully suppressed bladder activity. These studies highlight the clinical potential of TI electrical stimulation as a form of peripheral nerve stimulation.

#### 4.3.4 Applications of TIS in the retina

Due to its precise focusing characteristics, TIS shows promising applications in treating retinal degenerative diseases. Su et al. (2021) demonstrated that TIS could restore visual function in patients with retinal degeneration by electrically stimulating the retina through a computational modeling method. Their study showed that TIS gradually generates a localized electric field of increasing intensity in the retina as the acting electrodes move toward the posterior part of the eye (Su et al., 2021). Furthermore, the position of the convergence zone can be adjusted by modifying the current ratios of different electrode channels. This multipoint stimulation strategy allows for spatially selective retinal neuromodulation, making it a feasible method for targeted retinal stimulation in the presence of a certain degree of convergence and large areas. This suggests that the TI strategy could be a viable method for spatially selective retinal stimulation by effectively extending the stimulation area. Further research is necessary to validate the response of the retina to TIS, especially in studies targeting retinal diseases.

### 4.4 Other strategies

TIS with two different frequencies of 2 and 2.01 kHz could induce neuronal spiking activities in the hippocampus, with neurons in the neocortical regions being unaffected, however, relatively weaker TI currents were delivered to deep brain regions in the human head model compared to the murine model. A certain level of TI currents inevitably flowed through unwanted regions when using the conventional TIS even when the electrode conditions were optimized (Lee et al., 2020). To solve the issue, Lee proposed the multipair TIS with additional electrode pairs, in contrast to the conventional two-pair TIS, to effectively increase the focality of stimulation at the target and reduce the unwanted modulation of neocortical areas (Lee et al., 2022).

Recent studies have further advanced the focality control of TI. Botzanowski et al. (2025) demonstrated multipolar temporal interference (mTI) in non-human primates and rodents, utilizing multiple carrier frequencies to generate overlapping envelopes. Multipolar designs with overlapping interference envelopes (e.g., 8 frequencies generating 4 modulation regions) allow independent control of stimulation volume and intensity. This approach has successfully activated the superior colliculus in primates at depths unreachable by conventional noninvasive methods while simultaneously reducing cortical engagement. Computational modeling suggests mTI reduces off-target field strength by >50% compared to conventional dipole TI (Botzanowski et al., 2025). Concurrently, Savvateev et al. (2023) introduced a phase-modulated multipair, using three electrode pairs with a 180° phase-shifted field to cancel off-target configuration, reducing off-target effects in mouse prefrontal cortex while preserving target stimulation efficacy, as validated by fMRI. These methodological innovations address critical limitations of traditional TI regarding spatial specificity.

Current experimental results indicate that multipair/mulitipolar TI stimulation shows promise in more precisely targeting specific neural regions. Achieving optimal stimulation effects may represent a future trend. Optimizing TIS for efficacy and safety involves careful consideration of parameters such as electrode placement, frequency selection, current intensity, and stimulation patterns. Given the complexity of neural response to TIS, optimal electrode placement may vary depending on the patient’s anatomy and the targeted neural structures. A promising strategy could be to use a range of frequency pairs and adjust them according to the desired depth of stimulation and the physiological response of the patient. This would require real-time monitoring of neural activity, perhaps through techniques like EEG or fMRI, to obtain maximum efficacy. Further research, especially well-designed clinical trials, is needed to establish evidence-based guidelines for the optimal use of TIS.

## 5 Limitations and future directions

Significant progress has been made in noninvasive neuromodulation using TI stimulation, but several issues still require further investigation. Most current research focuses on computational modeling and numerical simulations, with many results validated only in rodent models. While acute TIS appears to be safe, long-term safety data, such as glial activation and thermal effects of high-frequency carriers, are lacking. Therefore, additional studies involving human subjects and extended observation periods are necessary to advance the field. The research on TI electrostimulation remains in its early stages, necessitating further exploration to elucidate the underlying mechanisms, optimize stimulation protocols, and explore potential therapeutic applications. Validating optimal TI stimulation parameters across different models is crucial, as anatomical differences significantly impact electric field distribution. Accurate modeling can help reduce discrepancies between simulations and real-world scenarios. Additionally, quantitative investigations into stimulation depth, intensity, and location, alongside multi-electrode methods and optimization algorithms, are essential for improving stimulation efficiency. The effects of carrier frequency, envelope frequency, and injected current magnitude on brain focusing also require further exploration. Understanding the neurophysiological mechanisms, potentially involving neurons, synaptic plasticity, cerebral blood flow, and glial cells, requires additional studies using neuronal models and animal experiments.

Assessing the safety and tolerability of TI electrical stimulation is fundamental if it is to be developed as a new therapeutic modality. Safety considerations include potential adverse effects on subjects and possible damage to deep brain structures and neurons. In one study, 100 subjects were evaluated for adverse effects of TI electrical stimulation at 2 mA, with only 4 experiencing mild effects such as fatigue and dizziness (Esmaeilpour et al., 2021). The electric field generated in the brain, less than 1 V/m, was within the safe range for currents of approximately 1–2 mA. Grossman et al. examined molecular mediators (e.g., neurons, glial cells, and synaptic molecules) to assess potential brain damage, confirming the safety of TI stimulation as no changes in the number or morphology of neurons or synapses were observed. However, the phenomenon of high-frequency conduction block with TI stimulation, potentially affecting off-target neurons and causing unwanted side effects, has been identified and requires attention (Mirzakhalili et al., 2020). While TIS has demonstrated safety in healthy populations (Piao et al., 2022), its application in individuals with implanted devices introduces unique risks that require careful consideration. The high-frequency alternating electric fields (~1–10 kHz) used in TIS may induce electromagnetic interference (EMI) with active implanted devices (e.g., pacemakers, responsive neurostimulators). Implanted devices with conductive materials (e.g., electrodes, wires) may distort the spatial distribution of TIS-generated fields, leading to unintended hotspots or reduced target engagement. Its application in patients with implanted devices demands rigorous risk assessment, patient-specific modeling, and device-tailored protocols. Collaborative efforts to standardize safety practices and advance monitoring technologies are critical to expanding TIS’s therapeutic potential.

Future research should focus on establishing safety limits for TI electrical stimulation and developing a comprehensive noninvasive stimulation system. Integrating the stimulation system with an acquisition system to form a closed-loop human brain conditioning system, capable of detecting stimulation sites and intensity, is essential. Computational modeling and animal experiments should be used to define safety criteria.

Noninvasive brain stimulation has been clinically available for diagnosing and treating brain disorders for decades (Walther and Baeken, 2021). TI electrical stimulation, as a novel modality, offers increased spatial specificity and depth selectivity compared to conventional techniques (Rampersad et al., 2019). Traditional noninvasive tES typically causes scalp pain and limits the strength of the injected current (Wu et al., 2021). In contrast, TI stimulation can selectively target specific brain regions, such as cortical and subcortical areas, avoiding scalp nerve irritation and pain (Vassiliadis et al., 2024b). TI holds promise for treating neurological and psychiatric disorders by targeting pathological brain circuits. It shows great potential for conditions such as Parkinson’s disease (Yang et al., 2024) and epilepsy (Missey et al., 2021). Theoretically, noninvasive magnetic induction TI, IF-tACS, and IS-tDCS methods have shown promising results. Magnetic induction TI stimulation offers better focus than TMS, and interferential pulsed tES methods are more effective than tES without affecting the cerebral cortex. Controlled experiments with TI should be performed to validate these improved methods and facilitate their wider application.

## 6 Conclusion

The aforementioned studies have shown that TIS can effectively penetrate the cerebral cortex and modulate neural activity in deep brain regions. The effects of TIS are influenced by multiple parameters, including electrode arrangement, stimulation intensity, and duration, with a positive correlation between these factors. Furthermore, TIS shows promise for treating a variety of CNS disorders, although the underlying mechanisms remain unclear. With advancements in noninvasive deep neuronal modulation, TIS is garnering increasing attention, and its application in clinical treatment appears both reasonable and effective. However, much remains to be learned through further research, as this technology is still in its infancy. Since its inception, TIS methods have primarily been used for modeling and animal experiments. The application of TIS in humans remains limited. Due to the heterogeneity of electrical properties across various brain and skull tissues, the authenticity and degree of scientific validation of stimulation parameters and effects derived solely from mathematical and biological modeling are significantly lower than those obtained from tissue modeling studies. Additionally, there is a lack of research applying this technique in animal models and clinical settings, indicating a need for further investigation at the mechanistic level. Consequently, there is still a long way for TIS to transition from the research phase to the application stage.

## Data Availability

The original contributions presented in the study are included in the article/Supplementary material, further inquiries can be directed to the corresponding author.
