# Supplementary material for: Advances in the application of temporal interference stimulation: a scoping review
Source: Front Hum Neurosci. 2025 May 30;19:1536906. doi: 10.3389/fnhum.2025.1536906 (PMC12162641; doi:10.3389/fnhum.2025.1536906)
Supplement: Supplementary file 1 [file Table_1.docx]

**Table S1. a detailed summary of included studies.**

| **Included study** | **Subjects** | **Target** | **Results** |
| --- | --- | --- | --- |
| **[Grossman et al., 2017](https://www.ncbi.nlm.nih.gov/pmc/articles/PMC9912300/#B29)**  **Noninvasive Deep Brain Stimulation via Temporally Interfering Electric Fields** | Mice | Hippocampus | TI electrical stimulation can successfully activated neurons in the hippocampus of mice without affecting neurons of the overlying cortex. |
| **Rampersad et al., 2019**  **Prospects for transcranial temporal interference stimulation in humans: A computational study** | Murine model and human model | Pallidum, hippocampus, motor cortex | tTIS is capable of producing more focal fields and allows for better steerability for neuromodulation of deep brain areas. |
| **Lee et al., 2020**  **Individually customized transcranial temporal interference stimulation for focused modulation of deep brain structures: a simulation study with different head models** | Three realistic and individual head models | Right hippocampus | Optimization of TI stimulation parameters allows for the delivery of the desired amount of TI current to the target region while effectively, reducing the TI currents delivered to cortical regions compared to the other stimulation approaches. |
| **Li et al., 2020**  **Analytical and Experimental Investigation of Temporal Interference for Selective Neuromuscular Activation** | Healthy volunteers | Three nerve/muscles that control human fingers | TI can selectively activate three nerves/muscles controlling human fingers. The experiment validates the feasibility of a selective neuromuscular stimulation approach. |
| **Song et al., 2021**  **Multi-channel transcranial temporally interfering stimulation (tTIS): application to living mice brain** | Mice | Motor cortex neurons | Multi-channel tTIS can improve the focality and reduce the sensation of discomfort; multi-channel tTIS can activate motor cortex neurons in living mice. The movement frequencies of the contralateral forepaw are consistent with the corresponding difference frequencies. |
| **Missey et al., 2021**  **Orientation of Temporal Interference for Non-invasive Deep Brain Stimulation in Epilepsy** | Male OF1 mice | The CA3 of the mouse hippocampus | An orientation-tunable form of temporally interfering (TI) electric fields can target the CA3 of the mouse hippocampus which focally evokes seizure-like events (SLEs) having the characteristic frequencies of rapid-discharges, but without the necessity of the implanted electrodes, and is demonstrated to strongly control the threshold for evoking SLEs. |
| **von Conta et al., 2021**  **Interindividual variability of electric fields during transcranial temporal interference stimulation (tTIS)** | Healthy volunteers | Left hippocampus, left motor area and thalamus | The electric field strengths inside the ROIs (left hippocampus, left motor area and thalamus) during tTIS are variable on single subject level. tTIS stimulates more focally as compared to tACS with much weaker co-stimulation of cortical areas close to the stimulation electrodes. Electric fields inside the ROI were, however, comparable for both tTIS and tACS. |
| **Song et al., 2021**  **Temporal Interference Stimulation Regulates Eye Movements and Neural Activity in the Mice Superior Colliculus** | Mice | Deep layers superior colliculus (SC) of mice | Temporal interference (TI) stimulation can evoke the neural activity in the deep layers of SC and the eye movements. The Granger causality (GC) analysis indicated that when the current exceeds 1 mA, the neural activity in deep layers of mice SC may cause the eye movements during TI stimulation. Changing the difference frequency of TI stimulation can also regulate the frequency of the deep layers neural activity and eye movements. |
| **Sunshine et al., 2021**  **Restoration of breathing after opioid overdose and spinal cord injury using temporal interference stimulation** | Rats | Diaghragm | TI can activate the phrenic neuromuscular system to sustain breathing after opioid overdose, preventing fatal apnea or activate spinal motor neurons following chronic cervical SCI. |
| **Collavini et al., 2021**  **Improvements on spatial coverage and focality of deep brain stimulation in pre-surgical epilepsy mapping** | Two realistic head models of drug-resistant epilepsy patients | Hippocampus and the Amygdala | Using contacts from different electrodes (x-DESM) may improve stimulation coverage and/or focality. The example of TI stimulation validated this method and demonstrated its potential application in DESM. |
| **Lee et al., 2021**  **An Efficient Noninvasive Neuromodulation Modality for Overactive Bladder Using Time Interfering Current Method** | 3D human and rat anatomical models; rats | Tibial nerve | Computational and in vivo experiments demonstrated that TIS has high penetration efficiency, thereby generating a stronger electric field than TENS. TIS applied to the tibial nerve of rats reduced contraction frequency and increased bladder voiding volume. |
| **Su., 2021**  **Computational Modeling of Spatially Selective Retinal Stimulation With Temporally Interfering Electric Fields** | An eyeball model | Retina | This study developed a computational model using multi-electrode configurations to demonstrate spatially selective retinal stimulation via TIS. By regulating the current ratio of different electrode channels, the position of the convergent region could be modulated. |
| **Ma et al., 2022**  **High Gamma and Beta Temporal Interference Stimulation in the Human Motor Cortex Improves Motor Functions** | Healthy human participants | Left primary motor cortex (M1) | TI stimulation with an envelope frequency of 70 Hz promoted the reaction time (RT) performance of the motor task compared with the sham condition in the RRTT experiment. TI stimulation with an envelope frequency of 20 Hz applied over M1 enhanced the motor learning performance compared with sham stimulation, and the performance was positively correlated with the MEP increase in the SRTT experiment. |
| **Zhu et al., 2022**  **Temporal Interference (TI) Stimulation Boosts Functional Connectivity in Human Motor Cortex: A Comparison Study with Transcranial Direct Current Stimulation (tDCS)** | Healthy young participants | Left primary motor cortex (M1) | TI and tDCS both increased resting-state functional connectivity between M1 and secondary motor cortex (premotor cortex and supplementary motor cortex), and the enhancement of functional connectivity may be related to motor functions. |
| **Acerbo et al., 2022**  **Focal non-invasive deep-brain stimulation with temporal interference for the suppression of epileptic biomarkers** | Mouse models of epilepsy and human cadavers | Hippocampus | TI stimulation at the beating frequency of 130 Hz is able to suppress epileptic biomarkers of epilepsy in a mouse model of epilepsy and can target a deep structure in the human brain (hippocampus) with a limited field in the surrounding cortex and structures. TI therapy could be tested for efficacy before a potential DBS device implantation. |
| **Botzanowski et al., 2022**  **Noninvasive Stimulation of Peripheral Nerves using Temporally-Interfering Electrical Fields** | Mice | sciatic nerve | Temporal interference nerve stimulation (TINS) can stimulate the sciatic nerve via muscle movement and electromyography (EMG) recording; TINS can be used in the clinic to deliver acute therapy to PNS targets; TINS can allow clinicians to test and titrate stimulation parameters to determine if patients are good candidates for a more invasive and permanent therapeutic bioelectronic intervention. |
| **Piao et al., 2022**  **Safety Evaluation of Employing Temporal Interference Transcranial Alternating Current Stimulation in Human Studies** | Healthy adults | Left primary  motor cortex (M1) | TI-tACS is safe and tolerable in terms of neurological and neuropsychological functions and adverse effects for use in human brain stimulation studies under typical transcranial electric stimulation (TES) conditions (2 mA, 20/70 Hz, 30 min). This study lays the foundation for future human studies and clinical studies with TI-tACS. |
| **Zhang et al., 2022**  **Temporal interference stimulation targeting right frontoparietal areas enhances working memory in healthy individuals** | Healthy young adults | Right frontoparietal region | There was no correlation between the severity of the reported side effects and the predicted type of stimulation that the participants received. Working memmory (WM) appeared to be only marginally improved by TI compared to tACS-sham, and this improvement was only observed under high-load cognitive tasks. There was not observed significant differences between TI and TI-sham or TI and tACS in all N-back tests. |
| **Jabban et al., 2022**  **Pig Ulnar Nerve Recording with Sinusoidal and Temporal Interference Stimulation** | A femal pig | The ulnar nerve | High-frequency TI stimulation waveforms result in significantly larger stimulation artefacts than those experienced with lower frequencies, leading to amplifier saturation. No correlation between TI stimulation and detected spikes in the neural recording was found. |
| **Carmona-Barrón et al., 2023**  **Comparing the effects of transcranial alternating current and temporal interference (tTIS) electric stimulation through whole-brain mapping of c-Fos immunoreactivity** | Rats | / | Temporally interfering electric fields results in a global decrease in neuronal activation. The evoked neuronal activation depression affects most brain structures and is strongest at the electrode coordinates, decreasing gradually with the increase in the distance from the current source. tTIS increased c-Fos immunoreactivity of blood vessels perivascular astroglia. |
| **Iszak et al., 2023**  **Why Temporal Inference Stimulation May Fail in the Human Brain: A Pilot Research Study** | Healthy adults | Muscles in the upper limb; retina; visual cortex | TIS stimulation of peripheral nerves has efficacy, but no stimulatory effect was found in the central nervous system. |
| **Missey et al., 2023**  **Obstructive sleep apnea improves with non-invasive hypoglossal nerve stimulation using temporal interference** | Mice and Obstructive sleep apnea (OSA) patients | Hypoglossal nerves | Temporal interference (TI) using a bilateral TI apparatus could elicit both behavioral (tongue movement) and electrophysiological (tongue CMAP) responses without invasively implanting a cuff electrode around nerves; TI stimulation was found to clinically reduce apnea-hypopnea events in a subgroup of female patients with obstructive sleep apnea. |
| **Kwak et al., 2023**  **Effect of temporal interference electrical stimulation on phasic dopamine release in the striatum** | Rats | Striatum (STr) | Both STr-applied and cortex-applied TIS with a delta frequency (2 Hz) modulates evoked phasic DA release in the STr. These findings demonstrate that TIS could influence the neurochemical modulation of the brain. |
| **Wessel et al., 2023**  **Noninvasive theta-burst stimulation of the human striatum enhances striatal activity and motor skill learning** | Computational modeling; healthy young and older participants | Striatum | tTIS can noninvasively stimulate the striatum of the human brain using computational modeling, functional magnetic resonance imaging studies and behavioral evaluations. Theta-burst patterned striatal tTIS increased activity in the striatum and associated motor network. Furthermore, striatal tTIS enhanced motor performance, especially in healthy older participants as they have lower natural learning skills than younger subjects. |
| **Violante et al., 2023**  **Non-invasive temporal interference electrical stimulation of the human hippocampus** | Healthy humans | Hippocampus | TI stimulation can focally modulate hippocampal activity and enhance the accuracy of episodic memories in healthy humans through through functional magnetic resonance imaging and behavioral experiments. |
| **Popa et al.,2023**  **Effects of hippocampal noninvasive theta-burst stimulation on consolidation of associative memory in healthy older adults** | Healthy older adults | Hippocampus | Theta-burst patterned temporal interference electric stimulation (tb-tTIS) can noninvasively modulate the hippocampus in older adults with a consequent impact on associative memory and can induce faster recall of previously encoded face-name pairs 24 hours after stimulation when compared to a control stimulation. |
| **Botzanowski et al., 2023** **Controlling focality and intensity of non-invasive deep brain stimulation using multipolar temporal interference in non-human primates and rodents** | Mice and monkeys | Superior colliculus | Multipolar temporal interference (mTI) provides enhanced focality control at depth in anesthetized mice and monkeys without compromising on stimulation intensity; mTI evoked targeted activity at depth in the superior colliculus of an awake macaque. |
| **Qi et al., 2024** **Temporally interfering electric fields brain stimulation in primary motor cortex of mice promotes motor skill through enhancing neuroplasticity** | Mice | primary motor cortex (M1) region | TI stimulation with an envelope wave frequency of 20 Hz (Δ f = 20 Hz) once a day for 20 min for 7 consecutive days significantly improved the motor skills of mice by enhancing neuronal excitability and plasticity. |
| **Zheng et al., 2024**  **Repetitive temporal interference stimulation improves jump performance but not the postural stability in young healthy males: a randomized controlled trial** | Healthy young adult males | M1 leg area | Repetitive TI stimulation over M1 leg area improved the vertical jump height of healthy adult males but did not alter anti-fatigue ability and dynamic postural stability. |
| **Liu et al., 2024**  **Temporal interference stimulation targets deep primate brain** | Monkeys and patients with motor disorders | Substantia nigra | TI stimulation was administered to patients with motor disorders, and improvements in specific tremor symptoms were observed. |
| **Vassiliadis et al., 2024**  **Non-invasive stimulation of the human striatum disrupts reinforcement learning of motor skills** | Healthy humans | Striatum | Striatal tTIS applied at 80 Hz abolished the benefits of reinforcement on motor learning and increased the  neuromodulatory influence of the striatum on frontal areas involved in reinforcement motor learning. |
| **Liu et al., 2024**  **Noninvasive Deep Brain Stimulation via Temporal Interference Electric**  **Fields Enhanced Motor Performance of Mice and Its Neuroplasticity**  **Mechanisms** | Mice | Primary motor cortex (M1) | TI stimulation with an envelope frequency of 20 Hz could obviously improve mice motor performance. |
| **Mojiri et al., 2024**  **Quantitative analysis of noninvasive deep temporal interference stimulation: A simulation and experimental study** | Hodgkin-Huxley (HH) neuron model; a rat | Left motor cortex | Specific parameters of TIS, such as carrier frequency and current range, optimized neuron spiking in model. The results of the spatial spectrum of the rat hand movement were consistent with the spectrum information of the simulation results. Steering  the interfering region to the left motor cortex leads to noticeable contralateral movement of the right hand. |

Abbreviations: TI = temporal interference, tTIS = transcranial temporally interfering stimulation, ROI = region-of-interest, tACS = transcranial alternating current stimulation, SCI = spinal cord injury, RRTT = random reaction time task, SRTT = serial reaction time task, MEP = motor evoked potential, DBS = deep brain stimulation, CMAP = compound muscle action potentials, DESM = depth-Electrical stimulation mapping, TENS = transcutaneous electrical nerve stimulation.
